# A new blind color watermarking based on a psychovisual model

**DOI:** 10.1186/s13408-020-00094-9

**Published:** 2020-10-23

**Authors:** Pascal Lefevre, David Alleysson, Philippe Carre

**Affiliations:** 1 Laboratoire XLIM CNRS UMR 7252, Universite de Poitiers, Bât. H1 - SP2MI, 11 Bd Marie et Pierre Curie, TSA 41123, 86073 Poitiers Cedex 9, France; 2 Laboratoire LPNC CNRS UMR5105, Université Grenoble Alpes, Bâtiment Michel Dubois, 1251 Avenue Centrale, 38400 Saint-Martin-d’Hères, France

**Keywords:** Metrical space, Robust watermarking, Psychovisual model, Lattice QIM

## Abstract

In this paper, we address the problem of the use of a human visual system (HVS) model to improve watermark invisibility. We propose a new color watermarking algorithm based on the minimization of the perception of color differences. This algorithm is based on a psychovisual model of the dynamics of cone photoreceptors. We used this model to determine the discrimination power of the human for a particular color and thus the best strategy to modify color pixels. Results were obtained on a color version of the lattice quantization index modulation (LQIM) method and showed improvements on psychovisual invisibility and robustness against several image distortions.

## Introduction

Nowadays, technologies related to multimedia are more accessible than ever, and it is easier for anyone to abuse unprotected contents. Watermarking has been one of the numerous multimedia security solutions for almost thirty years. A lot of interest has been dedicated to color image watermarking algorithms very early, which remains today a complex problem.

Literature contains numerous color watermarking algorithms. Earlier solutions proposed to apply classical grayscale strategy only on one of the three color channels. For example, in the past, [12, 20] proposed to select the blue channel in order to minimize perceptual changes in the watermarked image at the cost of a weaker robustness. Other contributions proposed to modify the intensity component [19] or the saturation component [10] in order to increase the robustness at the cost of a stronger embedding distortion.

Though there are some recent works on color image watermarking, the majority of the published works use grayscale images as host images for embedding the watermark information. Using colors, some works suggest that color images can be treated as grayscale information and each color component has to be processed in the same fashion separately (for example, [9]). Since color images contain more information than the grayscale counterparts, color image embedding has a different scale of impact; moreover, it is admitted that color watermarking method tradeoffs (robustness vs invisibility) can be improved by considering a vector approach. According to this strategy, some recent works such as [1] described such approach using a PCA decomposition, or introduced particular adapted vectorized strategy such as [16] that proposed a vector approach and modified wavelet coefficients computed from the RGB color space or color transform. Others use specific color transform: in [13] a quaternionic discrete cosine transform is proposed. However, the psychovisual invisibility of the mark is not directly addressed.

One step further towards better psychovisual invisibility is to take into account how the HVS deals with color distortions. In [5], a two-dimensional mark is embedded in the chromatic plane xy. This approach uses a histogram manipulation technique proposed in [8]. To improve the invisibility, color distances corresponding to embedding distortions are computed in the L*a*b* colorspace. Then, this method was improved in [6] by adding the mark directly into the L*a*b* colorspace. In other words, the embedding is processed in the color domain and takes advantage of the low sensitivity of the HVS to perceive small color differences.

This paper aims to develop a watermark embedding scheme that handles color images more efficiently taking care of the local color discrimination power of the human eye. So far, no-one has fully investigated the perception of color differences in the context of color watermarking. In our work, we propose a 3D quantization algorithm (Sect. 2) minimizing the perception of color differences of the HVS. It is based on a psychovisual model of photoreceptor dynamics in the human eye (Sect. 3). After explaining how to adapt a vector quantization method for color quantization, we show performance improvements thanks to some experimental results on psychovisual invisibility and robustness against various image distortions.

Before starting the presentation of our method, we introduce some elements concerning watermarking. There are several ways in which we can model a watermarking process. In this article, we use a model which is based on a communication-based view of watermarking. Watermarking is a process of communicating a message from the watermarking embedder to the watermarking receiver, and the cover image is the channel. In a general secure communication model, on one side we would have the sender which would encode a message using some kind of encoding key in order to be resistant to channel-related perturbation (for example, correcting code). Then the message would be transmitted on a communication channel, which would add some noise to the encoded message (in the watermarking context, it is the attack aspect). The resulting noisy message would be received by the receiver which would try to decode it using the decoding key to get the original message back. In this article, we use the quantization process. Every watermarking system has some very important desirable properties which are often conflicting. The first important property is the image fidelity. Watermarking is a process that alters an original image to add a message to it. We want to keep the degradation of the image’s quality to a minimum: it is the notion of distortion. This property can be influenced by a parameter of the watermarking process: the embedding rate which measures the ratio between the size of the watermark (bits) and the number of samples of the host (cover image). The second important property is the robustness which is crucial for most watermarking systems. The watermarked message is altered during its lifetime, for example, by transmission over a lossy channel (attacks). A robust watermark should be able to withstand many operations: additive Gaussian noise, compression, color operations, etc.

## Color quantization

In this section, we describe a general three-dimensional color quantization method in a three-channel colorspace. As set out in the introduction, the color watermarking process is based on lattice QIM. We briefly review this classical strategy.

### Lattice QIM (LQIM)

Quantization index modulation (QIM) was introduced by Chen et al. in [7] and later extended as the lattice QIM method. We propose to describe the version studied in [15]. To embed a binary information $m \in \{0, 1\}$, a quantizer $Q_{m}$ is used to send a host sample *x* on a coset $\Lambda _{m}$ of $\Delta \mathbb{Z}^{L}$ corresponding to bit *m*. *x* is a vector of dimension *L* whose values can be pixel values, DCT or DWT coefficients, etc. We have

1 $$ y = Q_{m}(x, \Delta ) = \biggl\lfloor \frac{x}{\Delta } \biggr\rfloor \Delta + (-1)^{m + 1}\frac{\Delta }{4} $$

*y* is the result of the quantization. Cosets are defined as follows:

$$ \textstyle\begin{cases} \Lambda _{0} = \Delta \mathbb{Z}^{L} - \frac{\Delta }{4}, \\ \Lambda _{1} = \Delta \mathbb{Z}^{L} + \frac{\Delta }{4}. \end{cases} $$

**Figure 1:**
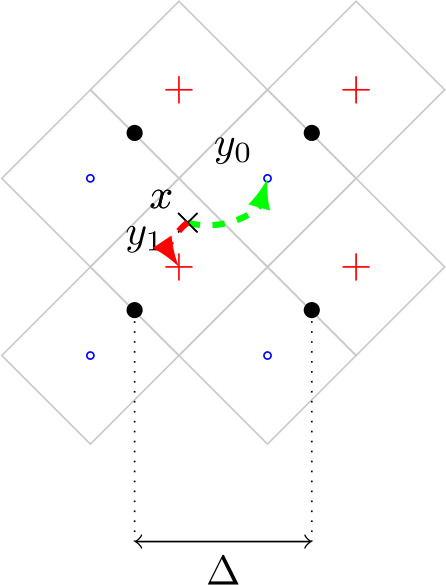
Quantization space 2D representation (Euclidean space of dimension $L = 2$). + (coset $\Lambda _{1}$) and ∘ (coset $\Lambda _{0}$) symbols represent bit 1 and 0 respectively

An example of quantization is depicted in Fig. 1 where a vector *x* is quantized to embed bit *m*. If $m = 0$, the result of the quantization is $y_{0} = Q_{0}(x, \Delta )$ and $y_{1} = Q_{1}(x, \Delta )$ otherwise.

The construction of the quantization space $\Lambda _{0} \cup \Lambda _{1}$ relies on the idea of equally spreading binary information in the Euclidean space so that any $x \in \mathbb{R}^{2}$ can be transformed into $y_{m}$ without important distortions. These distortions are controlled by the quantization step Δ. More formally, the distortion in dimension $L = 2$ is bounded so that

2 $$ \Vert y_{m} - x \Vert _{2} \leq \Delta \frac{\sqrt{2}}{2}. $$

Hence, one can note that a larger Δ leads to potentially more distortions in the image being watermarked.

At the detection step, one receives a vector $z \in \mathbb{R}^{L}$ that is modified from the original watermarked value $y_{m}$. In order to estimate bit *m*, one needs to find the closest coset $\Lambda _{m}$ (with respect to the Euclidean distance $\|\cdot\|_{2}$). In other words, we have

$$ \hat{m} = \mathop{\operatorname {arg\,min}} \limits _{m \in \{0, 1\}} \operatorname{dist} (z, \Lambda _{m}) $$

with

$$ \operatorname{dist} (z, \Lambda ) = \min_{y \in \Lambda } \Vert z - y \Vert _{2}. $$

Using Fig. 1 again, we plotted a diagonal grid (or cells centered with + and ∘) as a visual example in dimension $L = 2$. To illustrate the detection of *z*, it then suffices to identify the cell center $y_{m}$ where *z* is located.

### Color adaptation

For a given color pixel *P*, the embedding distortion is controlled by a direction vector $u_{P}$, i.e., the concept of scalar quantization is applied along the axis $u_{P}$.

The new value of the pixel after the watermarking by quantization (denoted by $P_{w}$) is obtained as follows:

3 $$ P_{w} = P + (s_{w}-s).u_{P} $$

with $u_{P}$ being a normalized direction vector depending on the color pixel *P*, $s = \langle P, u_{P}\rangle $ is the projection of the pixel vector *P* onto the direction vector $u_{P}$. $s_{w}$ is the modified scalar product by the quantification procedure introduced above:

$$ s_{w} = Q_{m(P)}(s, \Delta ) $$

with $m(P)$ being the message bit associated with the pixel *P*.

Let us note that this approach has some consequences on the color perception. Given a pair of pixels *P* and $P_{w}$ separated by a distance $d_{2}(P, P_{w})$, the perceived color distortion may be different for the HVS when considering a different pair of pixels $(P', P_{w}')$ separated by the same distance $d_{2}(P, P_{w})$.

At the detection step, $s_{w}$ is the coefficient to estimate in order to extract the embedded information:

4 $$ s_{w,a} = \langle P_{w,a}, u_{P_{w,a}}\rangle , $$

where $P_{w,a}$ is the watermarked and modified color pixel after some attacks and $u_{P_{w,a}}$ is its associated estimated direction vector. Consequently, to work properly, it is necessary that direction vectors $u_{P}$ and $u_{P_{w,a}}$ are close enough to correctly estimate $s_{w}$. If, as we will show later, the direction $u_{P}$ is given by a model of the direction of lower visibility, it is necessary for the psychovisual model to be stable. If the color difference $P_{w,a} - P$ is small (i.e., the color information of the cover image is not degraded by the watermarking process and the attacks), then $u_{P}$ and $u_{P_{w,a}}$ have to be closed.

The following section defines the general process to embed and detect a message into a color image. The key of the method is the setting of parameter $u_{P}$.

### Color direction embedding

We can use different strategies to choose a color direction vector $u_{P}$ for the embedding step. One of the aims is to find a direction that minimizes the color quantization noise for the HVS. An elementary approach consists in choosing a constant direction $u_{P}(P)=u$ for every color.

**Figure 2:**
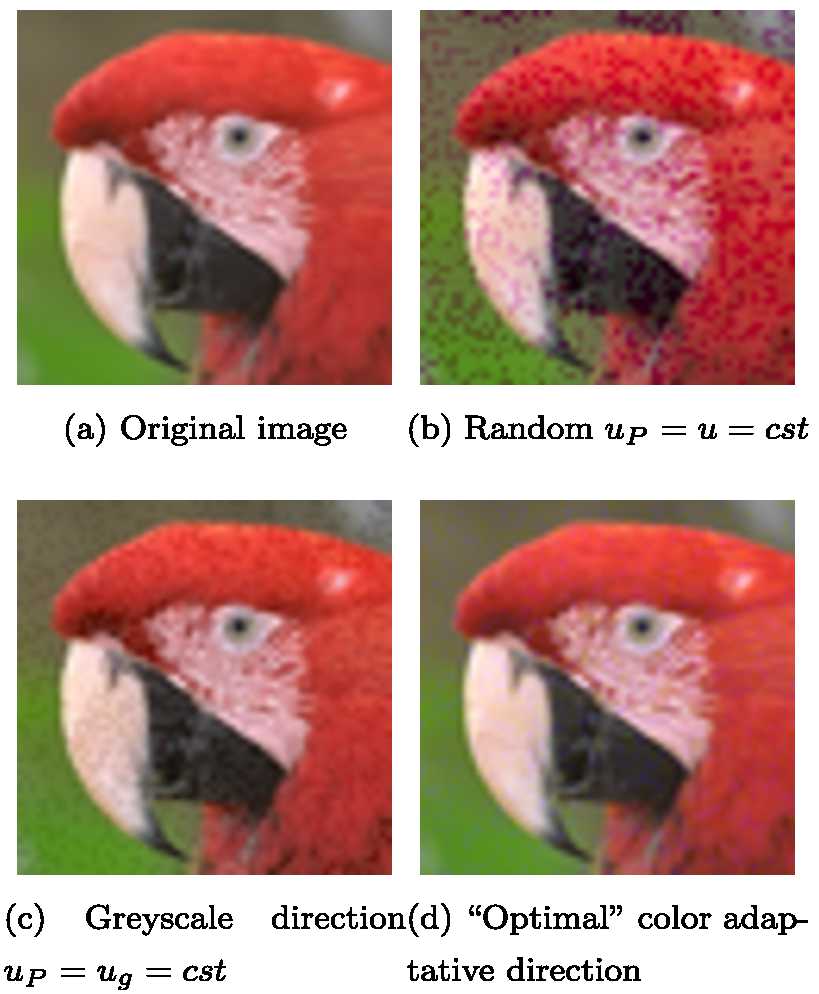
Examples of embedding with different strategies with constant numerical distortion. Images are cropped versions of *kodim23.png* from Kodak image database

To illustrate the influence of this choice, we randomly choose $u_{P}=u$ constant, then it is easy to perceive color distortions (Fig. 2b) because this direction introduces colors that are not relevant to the image content.

It is possible to decrease the perceived quantization noise by using, for example, the constant direction $u_{g} = (1, 1, 1)/\sqrt{(}3)$ associated with the grayscale or luminance information. This strategy is used by many authors. As illustrated in Fig. 2c, we experimentally concluded that $u_{g}$ produces a lower number of irrelevant colors. Compared to Fig. 2b, we can perceive fewer wrong colors for the same level of numerical distortion. However, this result is not totally satisfying for watermarking invisibility. Since the perception of quantization noise is different in function of the color information associated with the pixel, it is necessary to introduce an adaptive approach. For every color pixel *P*, a direction vector $u_{P}(P)$, depending on *P*, will be determined so that the color quantization noise is minimized for the HVS. A simple example using the *adaptive approach* is illustrated in Fig. 2d. We can see that the quantization noise is more difficult to see. This figure illustrates the main objective of the article: the use of the human visual system (HVS) model to improve the watermark invisibility.

In the next section, we propose a psychovisual model and a method to extract adaptive direction vectors required to set up the adaptive approach.

## Psychovisual approach to the HVS

Understanding how humans perceive color differences can allow us to minimize embedding distortions perceptually, i.e., improve the invisibility of a watermarking system. To achieve this goal, we require a model of color discrimination. This model is a simplification of the sophisticated neural process along the whole visual chain and solely involved photoreception layer. The model is three-dimensional because of human trichromacy, and it mimics the representation of light by cone receptors for their spectral sensitivities as well as their dynamics of intensity coding.

The model is an oversimplification of what is called neurogeometry which was proposed in the context of form perception based on achromatic information. A detailed description of the construction of forms by the primary visual cortex is described in [17, 18]. Few others have considered neurogeometry in the context of color vision [4, 11, 21].

**Figure 3:**
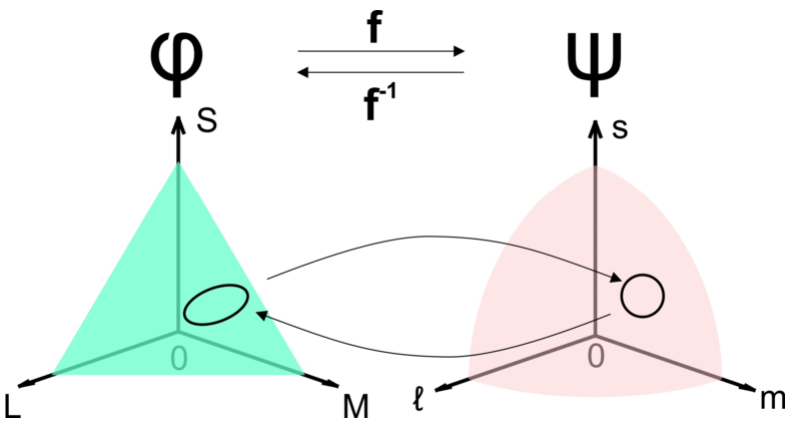
Mapping between physical to perceptual space. We suppose the visual system operates a non linear mapping **f** between the space of stimulus *φ* to the space of perception *ψ*. A circle of constant discrimination on an perceived isoluminant surface on ***ψ*** correspond to an ellipse in a physical isoluminant surface on *φ*

In the model we suppose that the visual system operates a nonlinear mapping between the space of physical stimuli *φ* and the perceptual space *ψ*. The map between those spaces is given by the neural function. This model is illustrated in Fig. 3. A circle of constant discrimination on a perceived isoluminant surface on ***ψ*** corresponds to an ellipse in a physical isoluminant surface on *φ*.

We will give an explicit nonlinear and parametric transform between *ϕ* and *ψ*. We made the hypothesis that the perceptual space *ψ* is an Euclidean space; consequently, contours of constant discrimination are spheres. Using classical tools of differential calculus one can endow the space *ϕ* with a metric obtained from the Euclidean metric on *ψ*.

Let us define **f** as a simplification of the Naka–Rushton function: the transduction of the light by the photoreceptor [3]:

5 $$\begin{aligned}& \begin{gathered} \mathbf{f}(x) = \alpha \frac{x}{x+1}, \\ y = \mathbf{f}(x/x_{0}) = \alpha \frac{x}{x+x_{0}} \end{gathered} \end{aligned}$$

with *y* being the transduction level, i.e., represents the electrical responses of a cone in function of *x* the excitation level of the cone produced by the light, $x_{0}$ is the adaptation state, and *α* is the gain of the cone channel. $x_{0}$ is modified in function of the average excitation level of the photoreceptor. Equation (5) represents a photoreceptor behavior in function of two constants *α* and $x_{0}$ fixed by environmental conditions and the HVS state. In fact, the adaptation process of a photoreceptor is only partially understood and this model is probably a simplification.

**Figure 4:**
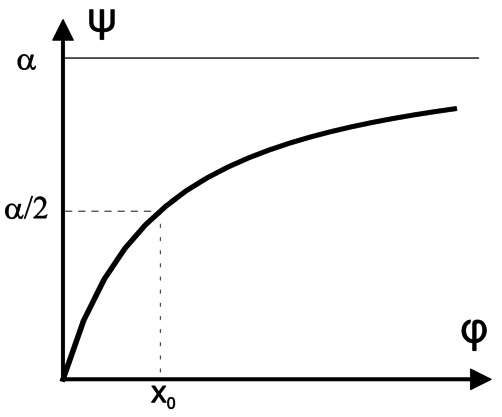
Nonlinear transduction function. In the model, the response of a photoreceptor *y* is a simplification of the Naka–Rushton function and writes: $y=\alpha x/(x+x_{0})$. This function saturates at *α* when $x \to \infty $. Also, $x_{0}$ defines half saturation value. The steepness of the graph depends on the value of the parameter $x_{0}$

The function saturates at *α* when $x \to \infty $ and $x_{0}$ gives the half maximum of the curve. Depending on $x_{0}$, the graph of the function changes. Low $x_{0}$ gives high steepness and high $x_{0}$ gives low steepness of the graph. This nonlinear function is illustrated in Fig. 4.

For the three-dimensional space of physical stimuli *φ*, we can write the correspondence between the physical space and the perceptual space as follows:

6 $$\begin{aligned} \begin{aligned} \mathbf{f} : \varphi &\to \psi \\ x &\mapsto y = \mathbf{f} (x )= \biggl( \frac{\alpha _{L} L}{L+L_{0}}, \frac{\alpha _{M} M}{M+M_{0}}, \frac{\alpha _{S} S}{S+S_{0}} \biggr), \end{aligned} \end{aligned}$$

where $x=(L,M,S)$ is the coordinate of the physical stimulus in *φ* space, $x_{0}=(L_{0},M_{0},S_{0})$ and $\alpha = (\alpha _{L}, \alpha _{M}, \alpha _{S})$ are respectively the adaptation of the observer and the gain of color channels. We implicitly assume here without lost of generality that the coordinates in *φ* correspond to the responses of the L, M, and S cones. Then $y=(\ell , m, s)$ is the encoding of the physical stimulus *x* by the visual system adapted to $x_{0}$ with gain *α*.

**Figure 5:**
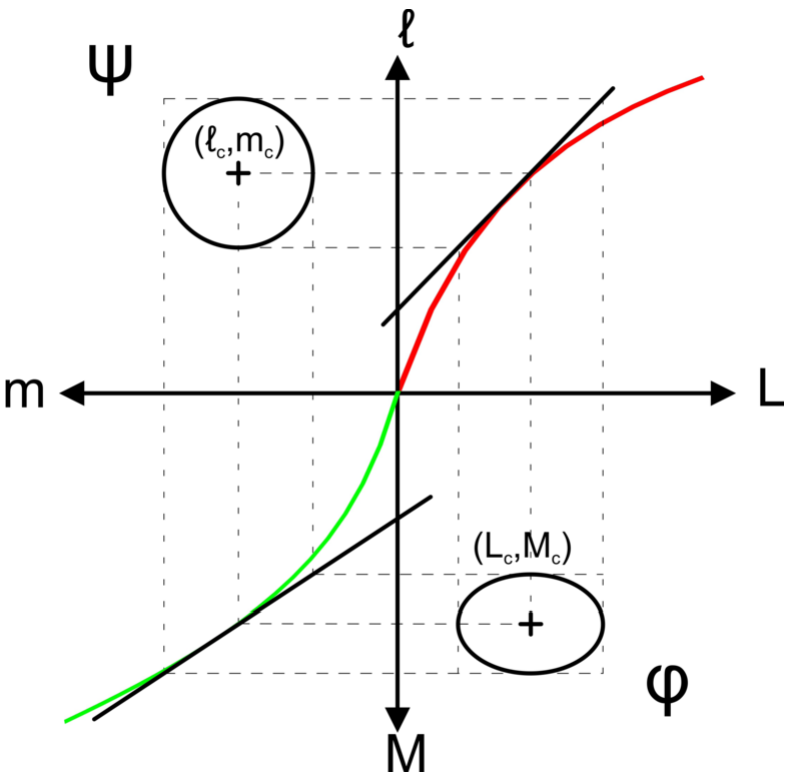
Transformation of a circle into an ellipse. A circle of constant discrimination in the perceptual space *ψ* corresponds to an ellipse in the space of physical stimuli *φ*. The deformation of a circle into an ellipse depends on the relative strength of the adaptation and gain factors on each color channel as well as the position in the space

According to this mapping, the metrics in the two spaces are related to each other. $g^{\phi }$ in the space of physical stimuli is given by the pull-back of the metric in the perceptual space $g^{\psi }$ through the mapping **f** and writes:

7 $$ g^{\varphi }(u,v) = g^{\psi }\bigl(D\mathbf{f}(u),D\mathbf{f}(v) \bigr), $$

where *u* and *v* are two vectors of the tangent space (Fig. 5) of *ψ* and *D***f** is the Jacobian matrix of **f** which writes:

8 $$\begin{aligned} D\mathbf{f}(x)= \begin{bmatrix} \frac{\alpha _{L} L_{0}}{(L+L_{0})^{2}} & 0 & 0 \\ 0 & \frac{\alpha _{M} M_{0}}{(M+M_{0})^{2}} & 0 \\ 0 & 0 &\frac{\alpha _{S} S_{0}}{(S+S_{0})^{2}} \end{bmatrix} . \end{aligned}$$

Because we consider the perceptual space to be Euclidean, the metric $g^{\psi }$ is the identity matrix, thus the metric $g^{\varphi }$ writes:

9 $$\begin{aligned} g^{\varphi }= \begin{bmatrix} \frac{\alpha _{L}^{2} L^{2}_{0}}{(L+L_{0})^{4}}& 0 & 0 \\ 0 & \frac{\alpha _{M}^{2} M^{2}_{0}}{(M+M_{0})^{4}}& 0 \\ 0 & 0 &\frac{\alpha _{S}^{2} S^{2}_{0}}{(S+S_{0})^{4}} \end{bmatrix} . \end{aligned}$$

We now propose to use this model in order to determine for each color of the image to be watermarked the direction optimizing the robustness/invisibility compromise.

## Application for color watermarking

In the previous section, we have introduced a color vision model to develop a more invisible watermarking method. In the LMS colorspace, we built ellipsoids that concretely illustrate how to control psychovisual distortion and then enable robustness improvements for a given psychovisual distortion level. Indeed, the notion of psychovisual distortions represents the color difference perception at embedding as opposed to numerical distortions. Then, since psychovisual distortions describe the watermark invisibility for the human better than numerical distortion measures, the hypothesis is that it becomes possible to obtain a better tradeoff between invisibility and robustness: for the same psychovisual distortions level, there are different levels of numerical distortions, and the choice of the more important numerical distortion is interesting for robustness improvements (maximizing numerical distortions increases robustness).

In other words, we propose in this section to extract from every color pixel *P* a direction vector $u_{P}$ which has the largest norm in its ellipsoid. $u_{P}$ is a vector in the RGB space where we have the maximum numerical distortions level allowed.

### Conversions

We give color space conversion matrices to work from psychovisual spaces XYZ and LMS to the RGB space. Transformations are chosen following the 1931 CIE standard.^1^ Conversion between color spaces is represented by the following matrices:

$$\begin{aligned}& M_{1} = M_{\mathrm{RGB} \rightarrow \mathrm{XYZ}} = \begin{pmatrix} 0.488718 & 0.310680 & 0.200602 \\ 0.176204 & 0.812985 & 0.010811 \\ 0.000000 & 0.010205 & 0.989795 \end{pmatrix}, \\& M_{2} =M_{\mathrm{XYZ} \rightarrow \mathrm{LMS}} = \begin{pmatrix} 0.38971 & 0.68898 & -0.07868 \\ -0.22981 & 1.18340 & 0.04641 \\ 0.0 & 0.0 & 1.0 \end{pmatrix}. \end{aligned}$$

Let $P_{\mathrm{RGB}}$, $P_{\mathrm{XYZ}}$, and $P_{\mathrm{LMS}}$ be color pixels in the respective spaces RGB, XYZ, and LMS. We have

10 $$ \begin{aligned} &P_{\mathrm{LMS}} = M_{2} P_{\mathrm{XYZ}}, \\ &P_{\mathrm{XYZ}} = M_{1} P_{\mathrm{RGB}}, \\ &P_{\mathrm{LMS}} = M_{2} M_{1} P_{\mathrm{RGB}}. \end{aligned} $$

Distortion properties discussed earlier can now be transferred through the space thanks to the conversion formulas. In the next subsection, we detail the direction vector extraction process.

### Direction vector extraction

In Sect. 2, we saw that the choice of a direction vector has a huge impact on watermark invisibility. When we choose a fixed direction for every color at fixed numerical distortions, we see strong psychovisual distortions in some areas of the image. However, the invisibility is significantly improved when the best direction vector is chosen for every color pixel. Since we suppose that psychovisual distortions are the same for all elements in ***ψ***, we can choose the furthest point from *P* denoted by $P_{f}$ to benefit from the maximum numerical distortions allowed at embedding to improve the robustness. Direction vector $u_{P}$ is defined so that $u_{P} = \overrightarrow{\mathrm{PP}_{f}}$.

We denote by $\mathcal{E}$ the set of points belonging to the perception volume associated with *P*. More precisely, $\mathcal{E}$ is the set of points in the RGB space associated with a constant discrimination. Then we have the furthest point $P_{f}$ from *P*:

11 $$ P_{f} = \max_{P' \in \mathcal{E}} \bigl\Vert P - P' \bigr\Vert _{2}. $$

A direct method of extracting an optimal direction with the based Jacobian estimation is to use the fact that the principal axes of the ellipses can be calculated from eigenvectors of the metric, and in this case the eigenvector associated with the lowest eigenvalue of the metric is $u_{P}$. To use this model, constants $L_{0}$, $M_{0}$, and $S_{0}$ and gains $\alpha _{L}$, $\alpha _{M}$, and $\alpha _{S}$ should be given. We use the parameter calculated in [2] for the MacAdam ellipses [14]: $(\alpha _{L}^{2} = 1665, \alpha _{M}^{2} = 1665, \alpha _{S}^{2} = 226)$, $(L_{0} = 66, M_{0} = 33, S_{0} = 0.16)$.

Finally, we deduce from equation (9) that the model is parametric, and to select the lowest eigenvalue (the value $\min [\frac{\alpha _{L}^{2} L^{2}_{0}}{(L+L_{0})^{4}}, \frac{\alpha _{M}^{2} M^{2}_{0}}{(M+M_{0})^{4}}, \frac{\alpha _{S}^{2} S^{2}_{0}}{(S+S_{0})^{4}}]$) and to give the expression of the direction $u_{p}$ for each color, the calculation shall be based on the parameters $\{ x_{0},\alpha ,M_{\mathrm{RGB} \rightarrow \mathrm{LMS}} \} $ and the studied pixel $P=(R,G,B)$.

In the last step, it is necessary to ensure that the detection step is possible after a modification of the marked image (we consider the case with acceptable damages). In other words, direction vectors computed at embedding and detection must be close enough to ensure an error free detection. The stability of the retrieval direction is linked with the color of the pixel and it also depends on the spatial environment of the pixel for some attacks (JPEG for example). To be robust, it is necessary that the direction of the watermarking between insertion and detection changes little or not at all. Given the model, this can happen when the color of the pixel is at the border of one of the three direction cases. In this case the color would have to be changed before watermarking to move away from this border. However, we see in the results of Sect. 5 that the robustness is not impacted and that it is not judicious to make the algorithm more complex.

## Psychovisual algorithm for color images

**Figure 6:**
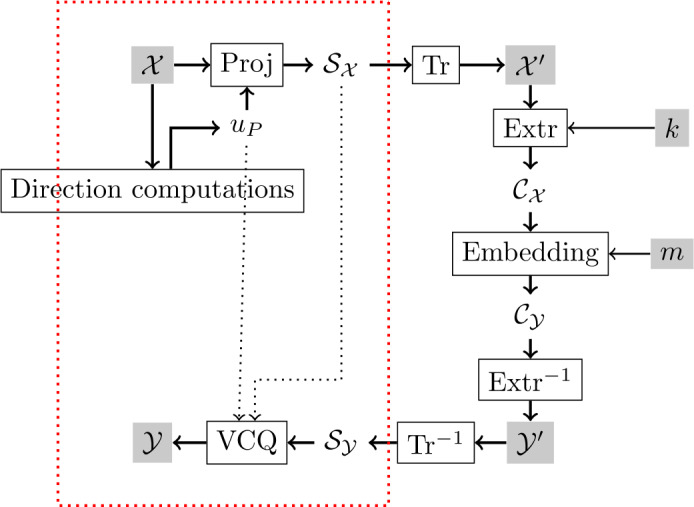
Classic embedding scheme combined with a vector color quantization method based on the psychovisual model. Steps in the red box represent the vector color quantization. Tr and Extr are space transforms and coefficient extraction respectively. *k* is the secret key and *m* is the embedded message

In this section, we introduce an embedding algorithm which improves the psychovisual quality of a marked image. Compared to the classic embedding scheme for grayscale images, we propose to add a step to adapt the embedding process for color images (Fig. 6).

Before choosing the quantization space, a direction vector $u_{P}$ is computed using the psychovisual model for every color pixel *P* of host image $\mathcal{X}$. A grayscale image $\mathcal{S}_{\mathcal{X}}$ is obtained by computing a scalar product for every *P* (Proj step in Fig. 6):

12 $$ s = \langle P, u_{P}\rangle . $$

**Figure 7:**
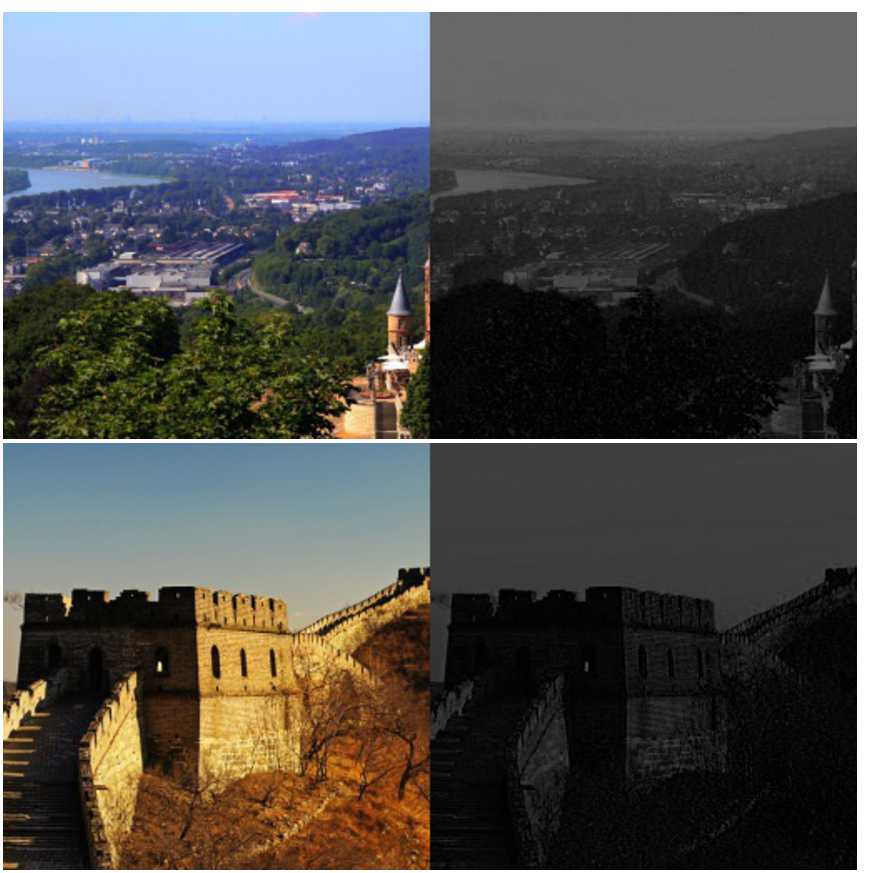
Image pairs ($\mathcal{X}$, $\mathcal{S}_{\mathcal{X}}$) randomly chosen from Corel image database

By considering the knowledge of computed direction vectors, we obtain a bijective relation between $\mathcal{X}$ and $\mathcal{S}_{\mathcal{X}}$. In Fig. 7, we can see examples of grayscale images $\mathcal{S}_{\mathcal{X}}$ obtained from $\mathcal{X}$.

The next step consists in choosing the quantization space (transform function denoted by Tr) of $\mathcal{S}_{\mathcal{X}}$ for coefficient extraction and embedding. For example, it is possible to apply directly the embedding process on the pixel value (the spatial domain) or to compute a discrete wavelet transform (DWT) of the image and apply the embedding process on the wavelet coefficient. In general, an image representation is chosen according to its properties and the robustness against some attacks (for example, it is well known that the wavelet domain is adapted to increase the robustness against compression).

Then, coefficients are extracted using the function Extr. In our work, we used a random selection of coefficients in the scalar image $\mathcal{S}_{\mathcal{X}}$. The extraction step is done in the watermarking space only accessible with the secret key *k* (only known to the embedder and the receiver).

Once coefficients $\mathcal{C}_{\mathcal{X}}$ are selected, they are modified by an embedding method, which is the lattice QIM in our case, according to the message *m*. The inverse transformation is performed on the modified scalar image $\mathcal{S}_{\mathcal{Y}}$. At last, using the following equation (VCQ), modified colors are computed, and we obtain the watermarked image $\mathcal{Y}'$ composed of modified pixels $P'_{w}$:

13 $$ P'_{w} = P + \bigl(s'_{w} - s\bigr)u_{P} $$

with $s'_{w}$ being the result of LQIM quantization when *s* is extracted.

**Figure 8:**
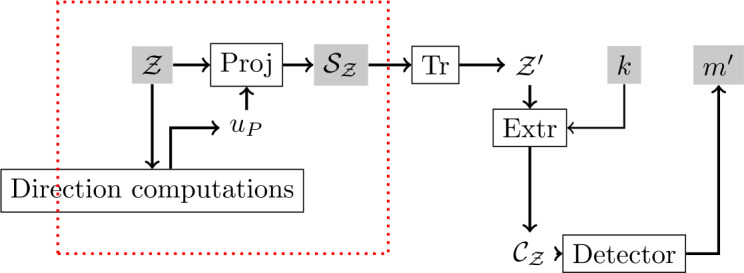
Classic detection scheme combined with the VCQ detection step (in the red box)

Detection step is illustrated in Fig. 8. Using key *k*, coefficients $\mathcal{C}_{\mathcal{Z}}$ are extracted from the scalar image $\mathcal{S}_{\mathcal{Z}}$ obtained from the received image $\mathcal{Z}$ thanks to equation (12). Then, a message estimation $m'$ is obtained with LQIM detector. If the attack is weak enough, then we have $m = m'$.

As we said before, the computation of direction vectors is very important for the detection process. Supposing colors are reasonably modified, the ability to successfully detect a message is guaranteed by the slow variation of direction vectors from one color to another. In our experiments, we show that this hypothesis is verified, and we obtain robustness improvements.

We have proposed a color quantization algorithm using a psychovisual model of the HVS to watermark color images. The lattice QIM method was chosen for our experiments but it is also possible to adapt other methods. The classic grayscale embedding and detection schemes can be adapted for color images by adding an extra layer containing the vector color quantization method proposed in Sect. 2. In the next section, we propose to evaluate invisibility and robustness performances.

## Experiments

For our tests, we used images from the Corel database where $1,000$ images are randomly chosen among the $10,000$ available. Image coefficients are randomly chosen. To evaluate the watermark invisibility, parameters such as the message length or the lattice dimension *L* are computed so that we obtain an adequate image quality and embedding rate.

### Psychovisual invisibility

In this subsection, we first propose to evaluate the psychovisual invisibility of several marked images with two methods denoted by GA and AA defined as follows:

- GA: color quantization with a constant direction vector $u = (1, 1, 1)$. *u* is the best choice in terms of invisibility for a constant direction vector approach according to our experiments.
- AA: color quantization using an adaptive axis $u_{P}$ in function of the color.

Both watermarking methods are modified versions of the LQIM method previously introduced in this article. For every method, direction vector norms are fixed to 0.5. The goal of this experiment is to show that the AA method has better psychovisual invisibility than the GA method.

In those experiments, we evaluate the numerical distortion with the help of a signal to noise ratio between the host image $\mathcal{X}$ and $\mathcal{Y}$ also known as DWR (document to watermark ratio).

**Figure 9:**
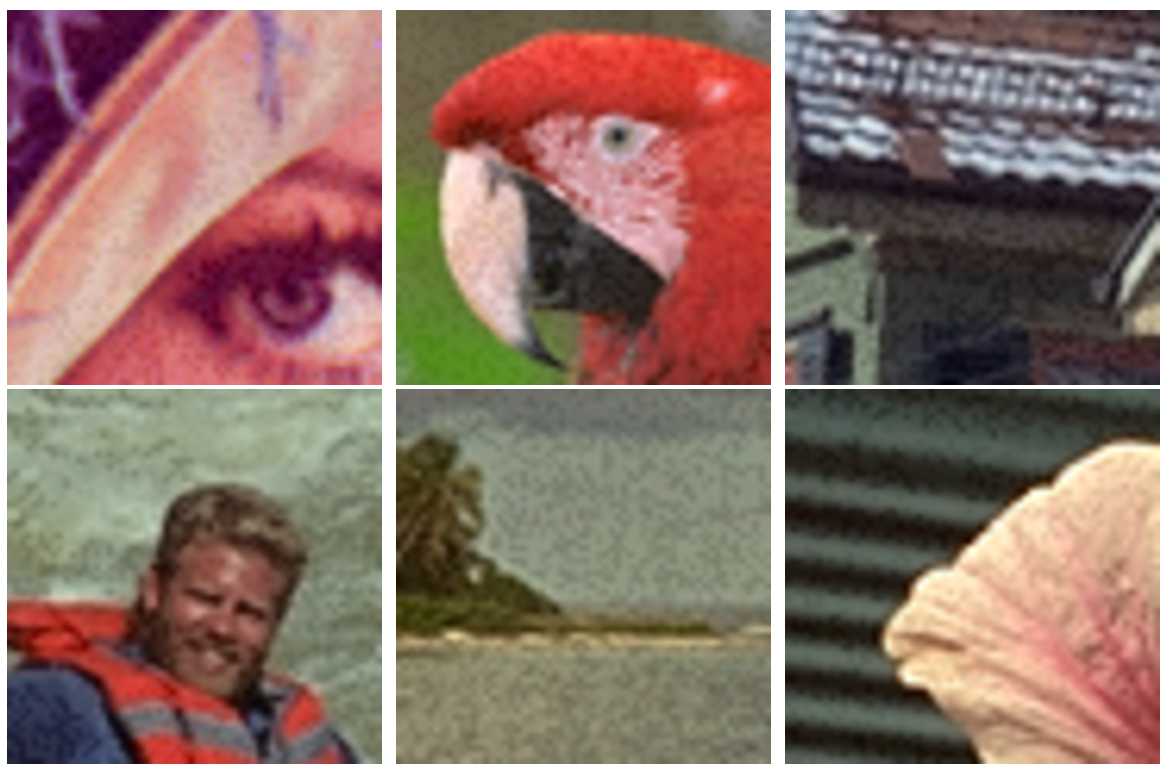
Cropped color images (Lenna and Kodak database images with size $60\times 60$) with the GA method. Average DWR is $\simeq -5.5\text{ dB}$ and ER =0.5

In Fig. 9, we show color image examples marked with the GA method. For each image, we easily see that colors are saturating towards gray. Then it is easier to perceive color differences with a naked eye. This can be explained by the random selection of quantized coefficients, which adds a salt and pepper noise texture to the image. Then the HVS will focus its attention on the noise by visual saliency.

**Figure 10:**
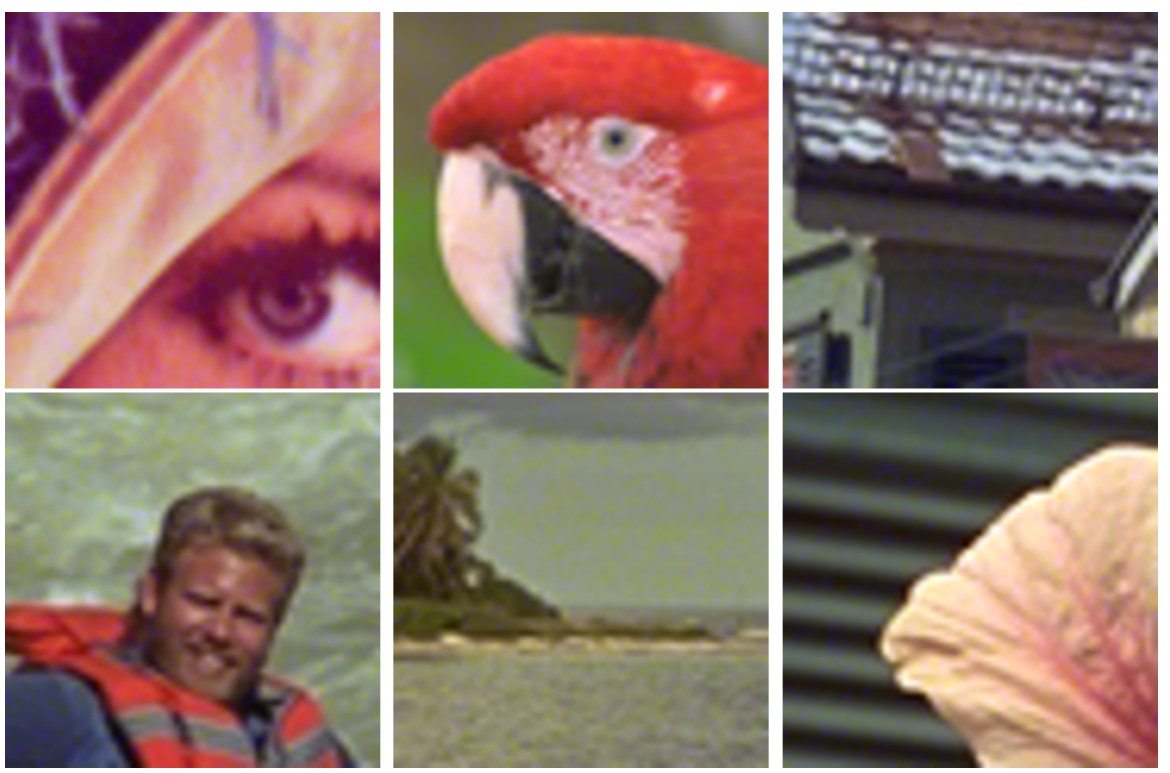
Cropped color images (Lenna and Kodak database images with size $60\times 60$) with the AA method. Average DWR is $\simeq -5.5\text{ dB}$ and ER =0.5

With the AA method (Fig. 10), the quantization noise makes colors saturate toward blue and green, i.e., it adapts itself in function of the color so it is harder to perceive it. Compared to pixels in homogeneous image regions, detecting a color difference is more difficult.

At equal numerical distortion level, marked images with both methods are not affected by the same psychovisual quantization noise. We observe in Figs. 9 and 10 that it is more difficult to perceive the psychovisual quantization noise with the AA method compared to the GA method.

For a better visual observation of the embedding noise, we have chosen a small image size of $60\times 60$ pixels with a strong embedding rate of 0.5. In practice, images are larger: for example, HD image size is $1080\times 1920$ and the embedding rate is smaller giving a more imperceptible quantization noise.

Secondly, to validate these visual observations, we have repeated this comparison experiment with 15 human test subjects. 24 images from the Kodak database with the size equal to $768\times 512$ have been selected. And, for each image, a pair of watermarked images with both methods are proposed to every test subject. Each observer has to decide which image (marked with GA and AA methods) is less noisy. Parameters are set to DWR = 20 dB, ER = 1/2 for each image.

Obtained results are given in Table 1. We can see that only 4% of images were described as less noisy in average with the GA method. Based on those results, we confirm the previous observation: watermarked images with the AA method provide better psychovisual invisibility than those with the GA method at the same numerical distortion level.

**Table 1:** Psychovisual experiments with marked image comparisons. This table shows the percentage of images described as less damaged for each method

Methods	GA method	AA method
Average votes	4% ± 3%	96% ± 3%

Now, we propose to analyze the robustness performances of both approaches at equal psychovisual distortion level.

### Robustness

#### Protocol

For our measures, we propose two embedding spaces for a message length of $n = 128$ bits, a lattice dimension $L = 2$, and the spatial domain is used (we modify directly the pixel value).

Before presenting the robustness results, we explain how to fairly compare GA and AA methods. First, we maintain satisfying psychovisual image quality/watermark invisibility for both methods. More precisely, we have experimentally determined the maximum average quantization step Δ before a mark becomes visible for each method and embedding domain (see Table 2). Notice that actually the threshold associated with the fact “the mark becomes visible” is set by a very simple method: the same image with different values of quantization step is proposed to some human observers, and each observer has to decide which image (marked with GA or AA methods) is degraded and which is not.

**Table 2:** Table of maximum quantization steps in average before a mark becomes visible

Δ/DWR	GA	AA
SP	16/29.4	24/38.8

In Table 2, we can see that we can use a greater quantization step with the AA method (more robustness allowed) compared to the GA method. We have an improvement of 5 dB from GA to AA. The AA method allows one to be more robust against several attacks.

We have measured the robustness performance of both methods using the quantization steps defined in Table 2: two average binary error rates (based on 100 embedding repetitions with random payloads and extractions) are computed in function of different attack strength parameters (±0.01). We propose now to analyze each result.

#### Contrast modification

We define the contrast modification by a multiplicative constant change of parameter *α*. Let *x* be a pixel value. We have

14 $$ y = \alpha x $$

with *y* being the modified version of pixel *x*. Errors are stronger when the difference between *α* and 1 is larger. This image processing also depends on the image content.

**Figure 11:**
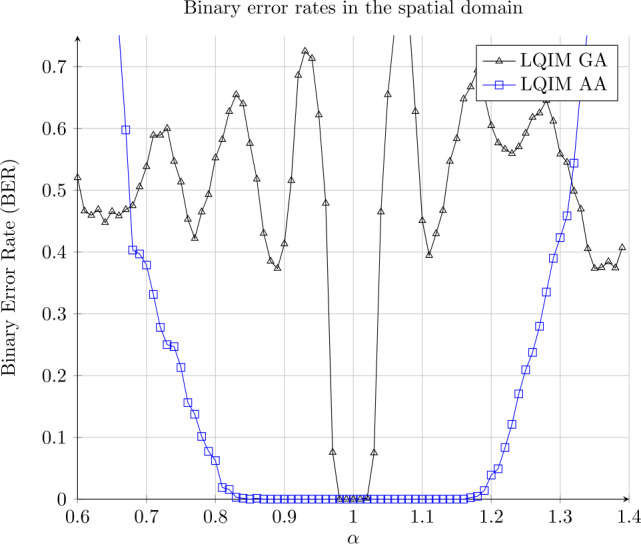
Binary error rate variations of GA and AA methods in function of a multiplicative constant *α*

Robustness results against this attack are illustrated in Fig. 11. There are more errors when *α* is not close to 1 and tends towards 0.5. We can see that LQIM AA curve is at 0 for a longer interval around value $\alpha = 1$ ($\alpha \in \{0.81, \ldots , 1.19\}$) than LQIM GA curve ($\alpha \in \{0.98, \ldots , 1.02\}$). In fact, LQIM AA is computed with a larger quantization step than LQIM GA.

#### Luminance modification

We model this attack by the following equation:

15 $$ y = x + \beta \times (1, \ldots , 1) $$

with *y* being the result of the luminance modification of a pixel value *x*.

**Figure 12:**
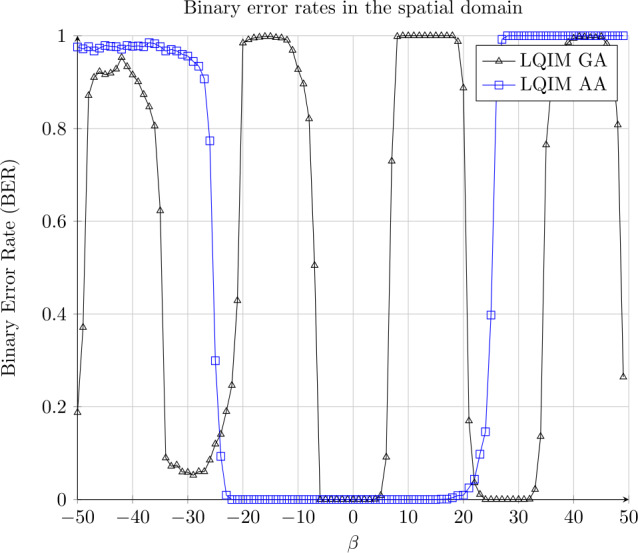
Binary error rate variations of GA and AA methods in function of an additive constant *β*

Results are shown in Fig. 12. In the spatial domain, error curves oscillate between 0 and 1 like a step function. This image processing is less dependent on the image content than the contrast modification. Again, LQIM AA is obtained with a larger quantization step than LQIM GA which explains why LQIM AA is at 0 for a larger interval.

#### Additive white Gaussian noise

**Figure 13:**
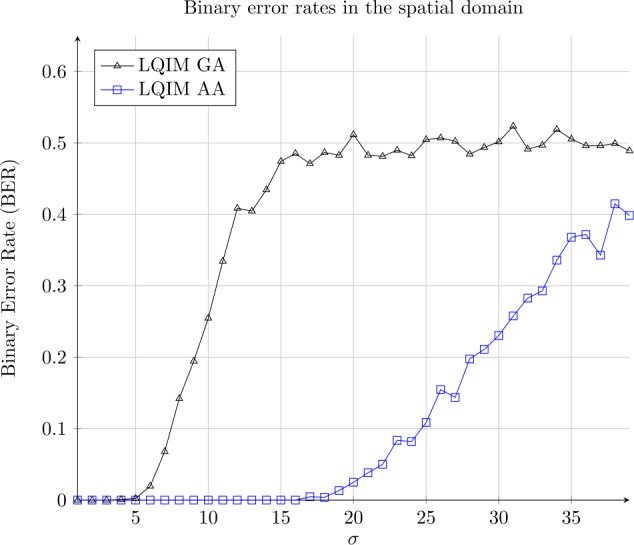
Binary error rate variations of GA and AA methods in function of the standard deviation parameter *σ* of an additive white Gaussian noise

We also made robustness experiments against additive white Gaussian noise (Fig. 13). This image processing modifies every pixel of an image with a random value following a Gaussian distribution and is parametrized by parameters $\mu = 0$ and *σ*. We can see that error rates are closer to BER =0 for LQIM AA than for LQIM GA.

#### Modifications of hue, saturation, and value

**Figure 14:**
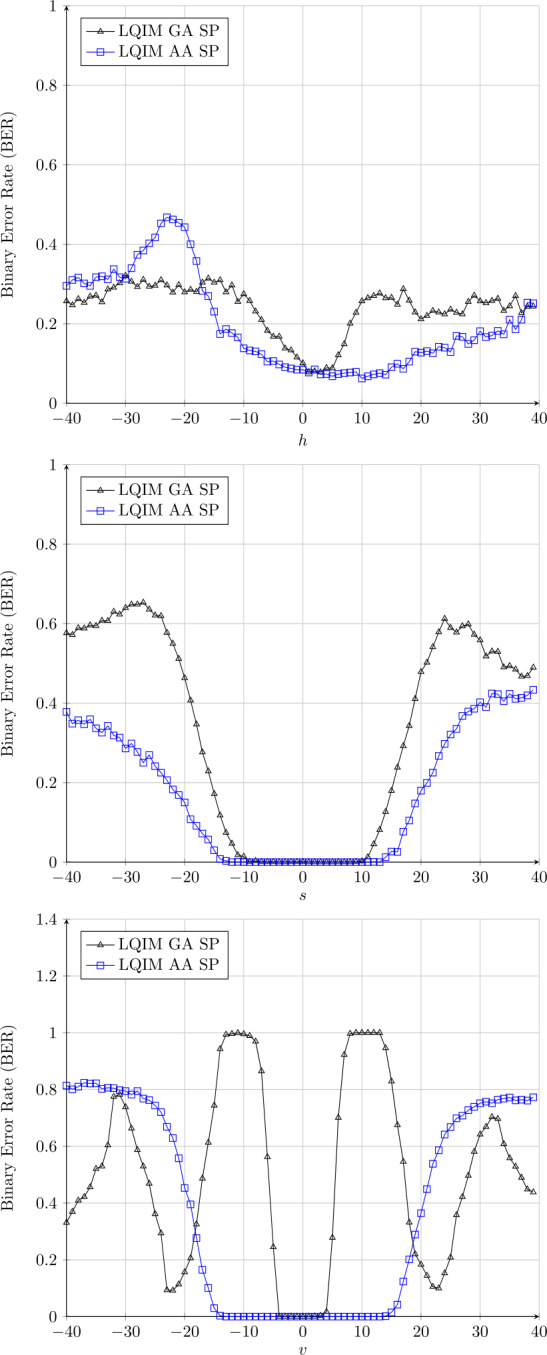
Error rate variations of GA and AA methods in spatial domains for the modifications of hue, saturation, and value

Finally, we propose to show results about HVS color space component modifications which produce color distortions and how this affects our watermarking methods. Watermarked images are converted in HVS space and color components *h*, *s*, and *v* are modified using the image library OpenCV (component variations between 0 and 255). Obtained results are illustrated in Fig. 14.

In this figure, we can see that LQIM AA SP curves are closer to BER =0 than LQIM GA SP curves.

Since the results are better with the adaptive strategy, we can deduce that the system fails to decode the message correctly when the pixel modification due to the attack becomes too large, although with the adaptive approach we were able to define cells for each larger word (for constant visual degradation). In this case the decoding of the bit becomes random.

To conclude, we propose to discuss a generalization that can be made for your process by using a wavelet decomposition. This first step changes the representation of an image in terms of variations, i.e., in frequency bands, and explains why both methods are robust in this embedding domain. Moreover, we can have better robustness results using this image decomposition compared to the spatial domain. To illustrate this fact, we propose an illustration with the JPEG attack. We choose to embed a watermark at level 2 in the diagonal details plane to obtain better detection performances. Compared to spatial domain, DWT coefficients are floating numbers, and it is important to note that this does not change the embedding algorithm.

#### JPEG compression

JPEG compression is a lossy compression algorithm for color images. It allows one to reduce the amount of data to encode an image in function of a quality factor. A high quality factor means that the image quality is close to its original version and a low factor means that the compressed image is strongly damaged but requires much less data. For color images, JPEG compression deletes in majority the color information.

In the spatial domain, error curve LQIM AA is close to 0.5: the spatial domain is not adapted.

**Figure 15:**
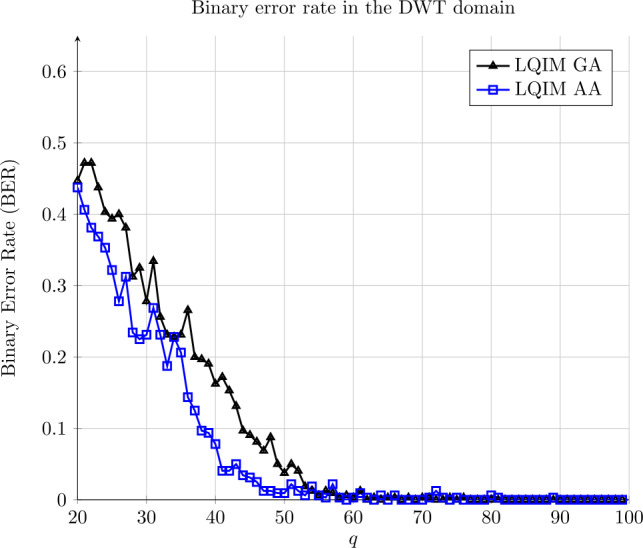
Binary error rate variations of GA and AA methods in function of a quality factor *q*

However, we observe a different result in Fig. 15 in the DWT domain. Error rates are rapidly reaching 0 with $q = 95$ for both methods. Modifying wavelet coefficients have mathematical properties that allow our methods to survive JPEG compression. Moreover, LQIM AA curve is reaching 0 faster than LQIM GA: we have a robustness improvement by working with wavelet coefficients.

## Conclusion

In this article, we proposed a color vector quantization algorithm for color image watermarking. It allows embedding watermark information along a direction vector in the RGB color space. Compared to an approach where the vector direction is fixed, our approach uses an adaptive direction calculated for every pixel in the image and provides better invisibility of the mark.

The adaptive method uses a Riemannian model of the representation of light by photoreceptors including their dynamical responses. Since the model is parametric, we use real experiment (MacAdam ellipses) to compute parameters. The model is then able to guess what is the direction of less discrimination around a color.

For a watermarking application, we used this model to extract direction vectors in order to minimize the perception of color differences at embedding and proposed to adapt the well-known lattice QIM method for color images.

Our experiments showed good improvements for the adaptive method in terms of psychovisual invisibility, compared to the method which uses a constant approach, at equal numerical distortions. After adjusting the invisibility/robustness tradeoff (comparing at equal psychovisual distortions), we compared the robustness of both methods against several attacks and concluded that the adaptive method is more robust than the constant method. Moreover, results were improved on both sides by using the wavelet transform as an embedding domain for particular attacks (like compression).

In this first study of watermarking invisibility using HVS model, we use a simple model taking into consideration only the encoding by photoreceptors and their dynamics. A natural extension of the model would be the use of a three-layer model accounting for the other cells (bipolar, ganglion) in the retina that also regulates light information. Indeed, it has been shown that the three-layer model allows a better account for the color discrimination data [3]. Another extension would be to use a pseudo-Riemannian model of color vision that preferentially describes color attribute such as hue, saturation, and brightness.

## Data Availability

Not applicable.

## Footnotes

1 White point of equal energy *E*.

## Abbreviations

HVS: Human visual system
LQIM: Lattice quantization index modulation
